# Epstein-Barr virus DNA load in chronic lymphocytic leukemia is an independent predictor of clinical course and survival

**DOI:** 10.18632/oncotarget.4418

**Published:** 2015-06-10

**Authors:** Carlo Visco, Erika Falisi, Ken H. Young, Michela Pascarella, Omar Perbellini, Giuseppe Carli, Elisabetta Novella, Davide Rossi, Ilaria Giaretta, Chiara Cavallini, Maria Teresa Scupoli, Anita De Rossi, Emanuele Stefano Giovanni D'Amore, Mario Rassu, Gianluca Gaidano, Giovanni Pizzolo, Achille Ambrosetti, Francesco Rodeghiero

**Affiliations:** ^1^ Department of Cell Therapy and Hematology, San Bortolo Hospital, Vicenza, Italy; ^2^ Department of Hematopathology, The University of Texas MD Anderson Cancer Center, Houston, Texas, USA; ^3^ Department of Microbiology, San Bortolo Hospital, Vicenza, Italy; ^4^ Section of Hematology, Department of Medicine, University of Verona, Verona, Italy; ^5^ Division of Hematology, Department of Translational Medicine, Amedeo Avogadro University of Eastern Piedmont, Novara, Italy; ^6^ Research Center LURM (University Laboratory of Medical Research), University of Verona, Verona, Italy; ^7^ Section of Oncology and Immunology, Department of Surgery, Oncology and Gastroenterology, University of Padova, Padova, Italy; ^8^ Istituto Oncologico Veneto(IOV)-IRCCS, Padova, Italy; ^9^ Department of Pathology, San Bortolo Hospital, Vicenza, Italy

**Keywords:** Epstein-Barr virus, chronic lymphocytic leukemia, CLL, EBV DNA

## Abstract

The relation between Epstein-Barr virus (EBV) DNA load and clinical course of patients with chronic lymphocytic leukemia (CLL) is unknown. We assessed EBV DNA load by quantitative PCR at CLL presentation in mononuclear cells (MNC) of 220 prospective patients that were enrolled and followed-up in two major Institutions. In 20 patients EBV DNA load was also assessed on plasma samples. Forty-one age-matched healthy subjects were tested for EBV DNA load on MNC. Findings were validated in an independent retrospective cohort of 112 patients with CLL. EBV DNA load was detectable in 59%, and high (≥2000 copies/µg DNA) in 19% of patients, but it was negative in plasma samples. EBV DNA load was significantly higher in CLL patients than in healthy subjects (P < .0001). No relation was found between high EBV load and clinical stage or biological variables, except for 11q deletion (P = .004), CD38 expression (P = .003), and *NOTCH1* mutations (P = .05). High EBV load led to a 3.14-fold increase in the hazard ratio of death and to a shorter overall survival (OS; P = .001). Poor OS was attributable, at least in part, to shorter time-to-first-treatment (P = .0008), with no higher risk of Richter's transformation or second cancer. Multivariate analysis selected high levels of EBV load as independent predictor of OS after controlling for confounding clinical and biological variables. EBV DNA load at presentation is an independent predictor of OS in patients with CLL.

## INTRODUCTION

Epstein-Barr virus (EBV) is a γ-herpesvirus harbored by the great majority of adults worldwide, resulting in life-long infection [1]. Following primary infection, EBV remains latent in memory B-cells, and confined by EBV-specific cytotoxic T lymphocytes in the healthy host [2]. However, in patients with profound immunosuppression due to T-cell function impairment and/or insufficient antibody production, the infection may induce lytic replication of the EBV genome and B-cell proliferation. There is compelling evidence for an etiologic role of EBV in certain B-cell tumors including endemic Burkitt lymphoma [3, 4], post-transplant lymphoproliferative disease [3, 4], HIV-related lymphomas [5], and aggressive lymphomas of the elderly [6].

Chronic lymphocytic leukemia (CLL) is typically characterized by immunosuppression already manifest in the early phases of the disease [7]. Although CLL is not considered an EBV associated disease, CLL cells express the complement receptor that serves as EBV receptor [8]. Pre-clinical data indicate that CLL cells are difficult to grow after exposure to EBV [9, 10], although this can be achieved following cytokine activation [11] or by successful outgrowth of *in vivo* EBV infected CLL cells [10]. A recent epidemiological study has reported an association between EBV infection and risk of developing CLL [12]. Tarrand et al. [13] reported that LMP1 mRNA levels were higher in CLL patients than in healthy subjects (14% *vs*. 1% of healthy controls), suggesting that EBV late gene expression does occur at least in a subset of CLL cells. The same group reported that 38% of CLL patients had evidence of EBV infection by in situ hybridization for EBV EBER1, a small noncoding RNA species [14]. Furthermore, EBV infection correlated with accelerated clinical course, including Richter's transformation [14-16]. Another study showed a relation between latent EBV infection and CLL cases expressing *IGHV4-34* B-cell receptor configuration [17].

With this study we show that EBV DNA load obtained from mononuclear cells (MNC) of patients with CLL at disease presentation is higher than in healthy controls. Increasing levels of EBV DNA load were significantly associated to shorter survival.

## RESULTS

### EBV DNA load in MNC, sorted B-cells and plasma of patients with CLL, and in MNC of healthy subjects

EBV DNA load was detectable by real-time PCR (>0 copies/µg DNA) in 129 of the 220 patients (59%), and was high (≥2000 copies/µg DNA) in 43 of the 220 patients (19%). Distribution of EBV DNA levels among patients was skewed to the right and had a peaked distribution (skewness 4.906, Kurtosis 33.160 by K–S test). Mean and median value among patients were 1625 and 192, respectively (standard deviation 3877; range 0-36449). In all five patients tested in parallel in sorted B-cells, EBV DNA load gave similar results to that observed in MNC (± 30 copies/µg DNA).

Twenty patients (10 with EBV DNA load ≥2000, and 10 with <2000 copies/µg DNA) were tested for EBV DNA load on plasma samples. All 20 resulted negative.

**Figure 1:**
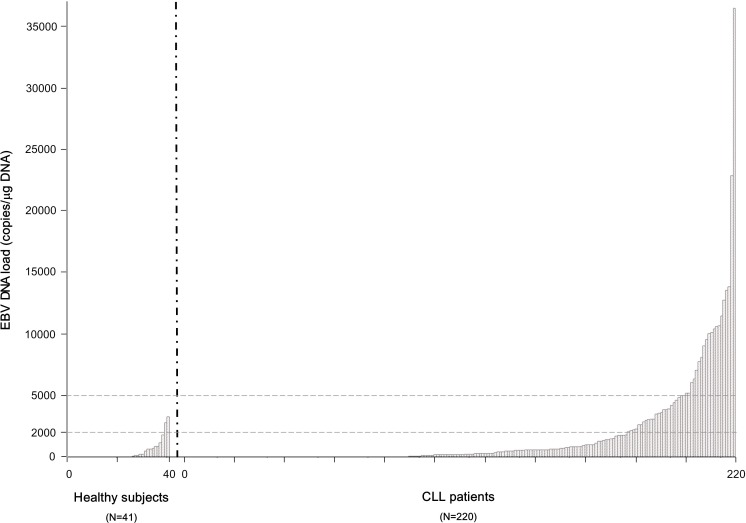
EBV DNA load measured as continuous variable in 41 healthy subjects compared to 220 patients with CLL Each bar of the histogram corresponds to a tested patient. EBV DNA was detectable by real-time PCR (>0 copies/µg DNA) in 129 (59%) (patients with CLL), and in 15 (37%) healthy subjects (P < .0001).

Viral load was significantly higher in patients with CLL than in healthy subjects (median value 0 copies/µg DNA; range 0-3234; P < .0001), as shown in Figure 1.

EBV DNA load was not associated with any of the EBV serological patterns (IgG+/IgM− versus IgG+/IgM+ versus IgG−/IgM−).

### Patients characteristics according to EBV status

Clinical and biological characteristics of our patients, divided according to EBV DNA load as a dichotomous variable, are listed in Table 1. Basically, a direct association between viral load, CD38 expression (P = .003), presence of del11q (P = .004), and borderline with mutations of *NOTCH1* (P = .05) was identified. There was a non-significant trend (P = .09) for older age in patients with high EBV DNA load.

Overall, 36 of 137 patients (26%) from the learning set had stereotyped B-cell receptor configuration. Of them, five had EBV DNA load ≥ 2000 copies/µg DNA, and 31 had EBV DNA < 2000 (22% *vs* 27%, respectively, P = .58). Subsets distribution appeared not biased between patients with high or low EBV DNA load.

**Table 1:** Clinical and biological characteristics of 220 patients with chronic lymphocytic leukemia at disease presentation, then divided according to EBV DNA viral load (copies/µg DNA)

	All Pts *N* = 220	EBV DNA ≥ 2000 *N* = 43	EBV DNA < 2000 *N* = 177	P-Value
**Median age**, years (range)	65 (30-86)	66 (47-86)	65 (30-83)	0.09*
**Female gender**	74 (34%)	19 (44%)	55 (31%)	0.10
**Median lymphocyte count**, ×10^3^/mmc (range)	9.1 (3.5-345)	8.1 (3.5-345)	10 (4.8-296)	0.24*
**Richter's transformation**	8 (4%)	3 (7%)	5 (3%)	0.17
**Second cancer**	11 (5%)	3 (7%)	8 (5%)	0.47
**BINET stage**				
A	171 (78%)	32 (74%)	139 (78%)	0.56
B	35 (16%)	9 (21%)	26 (15%)	0.31
C	14 (6%)	2 (5%)	12 (7%)	0.60
**FISH**°				
Normal	68 (39%)	11 (33%)	57 (41%)	0.43
del13q	64 (37%)	10 (30%)	54 (39%)	0.37
12+	13 (8%)	3 (9%)	10 (7%)	0.48
del11q	15 (9%)	7 (21%)	8 (6%)	**0.004**
del17p	5 (3%)	1 (3%)	4 (3%)	0.95
**IGHV mutational status**°				
Unmutated IGHV	79 (41%)	20 (53%)	59 (38%)	0.10
**Immunophenotype**				
CD38+	63 (29%)	21 (49%)	42 (24%)	**0.003**
ZAP70+°	92 (53%)	22 (61%)	70 (51%)	0.30
CD49d+°	49 (35%)	10 (36%)	39 (35%)	0.95
**Gene mutations**°				
*P53*	11 (6%)	1 (3%)	10 (6%)	0.236
*NOTCH1*	9 (4%)	4 (10%)	5 (3%)	**0.05**
*SF3B1*	14 (7%)	2 (6%)	12 (8%)	0.73
*MYD88*	6 (3%)	1 (3%)	5 (3%)	0.95
*BIRC3*	4 (3%)	0 (0%)	4 (3%)	0.35

Abbreviations: FISH: fluorescence in situ hybridization according to the hierarchical risk model; del13q: deletion in chromosome 13q14; del11q: deletion in chromosome 11q23; del17p: deletion in chromosome 17p12; +12: trisomy 12; IGHV: immunoglobulin heavy chain variable region genes.

* Calculated with the Mann-Whitney test; °FISH available in 173 patients, IGHV mutational status in 192, immunophenotype for ZAP70 in 172, CD49d in 139, *P53* mutations in 207, *NOTCH1* mutations in 204, *SF3B1*, *MYD88, and BIRC3* mutations in 193.

### Overall survival and time to first treatment

**Figure 2:**
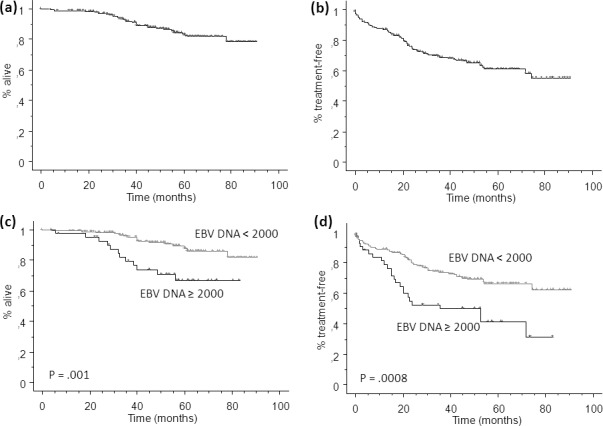
Kaplan Meier plot for overall survival (OS, a) and time to first treatment (TTFT, b) of patients from the learning set, dichotomized according to high or low EBV DNA load (≥ *vs* < 2000 copies/µg DNA, c and d) Hazard ratio and 95% confidence interval were 3,149 (1,486-6,672) and 2,243 (1,378-3,648) for OS and TTFT, respectively.

With a median follow-up of 54 months (range 12-98), we registered 30 deaths in the learning set. Median OS has not been reached, and 5-years OS was 83% ± 3% (Figure 2a). Patients with high EBV DNA load had a significant inferior OS (67% ± 8% at 5-years) than patients with low EBV DNA load (88% ± 3%, P = .001, Figure 2c). Two-years TTFT was 74% ± 3% (Figure 2b). Patients with high EBV DNA load had a significant inferior 2-years TTFT (52% ± 7%) than patients with low EBV DNA load (79% ± 3%, P = .01, Figure 2d). This predictive effect on TTFT was confirmed when the 171 patients with were analyzed separately (2-years TTFT 99% ± 1% for patients with low EBV DNA load *versus* 90% ± 5% for patients with high EBV DNA load, P = .001).

No significant difference was observed both in terms of OS and TTFT in patients with 0 copies/µg of DNA and those with <2000 copies/µg DNA, suggesting that the 2000 cut-off might be appropriate for defining high values. No impact of different front-line treatment approaches was evident on the EBV DNA predictive power.

Among other prognostic variables, univariate analysis recognized Binet A *vs* B or C (*P* < .0001), CD38, ZAP70, and CD49d expression (P = .002, P = .009, P = .01, respectively), *IGHV* mutational status (*P* < .0001), presence of del17p/*P53* gene mutations (P = .0005), and *NOTCH1* gene mutations (P = .03), as significantly related to TTFT. In terms of OS, all these variables maintained prognostic significance in univariate analysis except for ZAP70, CD49d and CD38 expression (the latter being borderline, P = .06), *NOTCH1* mutations (P = .14), and Binet stage (P = .18).

Mutations of *SF3B1, MYD88*, and *BIRC3* did not reach statistical significance due to the low number of mutated patients at CLL diagnosis in this prospective cohort of patients (Table 1).

### Validation set and cause of deaths

EBV DNA was detectable in 62 of 112 CLL patients (55%), and was ≥ 2000 copies/µg DNA in 22%. As reported in Table S1, similarly to the learning set, EBV DNA load was significantly associated with CD38 expression.

The predictive value of EBV DNA load was confirmed in the validation set, both in terms of OS and TTFT (Figure S1).

We observed 30 deaths in the learning set, and 32 in the validation set. Causes of death were apparently similarly distributed among patients with different EBV DNA load at CLL diagnosis. Second cancer was the cause of death in 5% and 12%, CLL progression in 42% and 41%, infection in 8% and 9%, age-related complications in 13% and 14% of patients with low and high EBV DNA load, respectively. None of the differences was statistically significant.

### Multivariate analysis

All significant variables from the univariate analysis comparisons were included in the multivariate analysis. Mutations of *NOTCH1* were not included because of the low prevalence of mutated samples. In order to avoid the possible confounding effect derived by the choice of different cutoffs for EBV DNA load, we computed this variable as continuous measurement.

In terms of OS, EBV DNA load resulted as the strongest independent predictive factor (P = .0004), together with *IGHV* mutational status (P = .02). Del17p/*P53* mutations were not significant in multivariate analysis due to their rarity (only 2 patients with del17p/*P53* mutation and high EBV DNA load, Table 1). The analysis identified Binet stage C (P = .0001), unmutated *IGHV* (P = .0005), and EBV DNA load as significant variables which independently predicted a shorter TTFT. Details are listed in Table 2.

**Figure 3:**
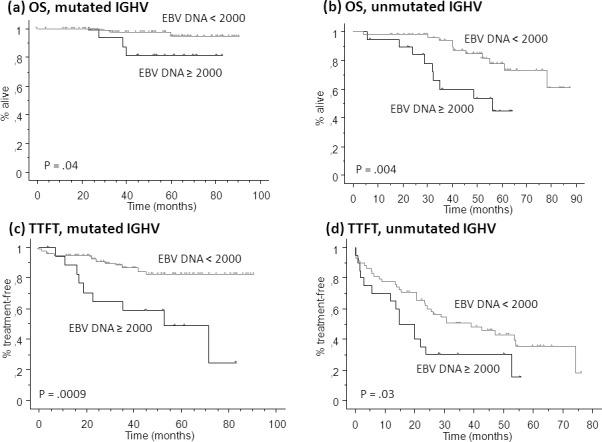
Kaplan Meier plot for overall survival (OS, a and b) and time to first treatment (TTFT, c and d) in patients of the learning set with mutated (a and c) or unmutated (b and d) immunoglobulin heavy chain variable region (*IGHV*) mutational status

In accordance with Cox's model results, a high EBV DNA load significantly predicted for poor OS and TTFT in both *IGHV* mutated and unmutated patients (Figure 3). The Cox model confirmed the independence of EBV DNA load and *IGHV* mutational status also in the validation set (hazard ratio 2.738, P = .005; hazard ratio 3.484, P = .0007, respectively).

**Table 2:** Multivariate Cox's regression analysis for (a) time to first treatment and (b) overall survival

(a)		
Parameter	HR (CI 95%)	P-value
Binet B or C	6.711 (2.457-18.518)	**0.0001**
CD49d positive	2.047 (0.977-4.285)	0.05
CD38 positive	1.152 (0.547-2.452)	0.71
Unmutated IGHV status	3.225 (1.568-6.622)	**0.0005**
del17p/P53 mutation	1.912 (0.814-4.498)	0.13
EBV DNA load	2.853 (1.242-5.239)	**0.002**

(b)		
Parameter	HR (CI 95%)	P-value
Unmutated IGHV status	3.215 (1.121-9.259)	**0.02**
del17p/P53 mutation	2.136 (0.684-6.671)	0.19
EBV DNA load	5.690 (2.173-14.905)	**0.0004**

Abbreviations: HR: Hazard Ratio; CI: Confidence Interval; *IGHV*: immunoglobulin heavy chain variable region genes; del17p: deletion in chromosome 17p12.

### EBV DNA load as continuous variable

**Figure 4:**
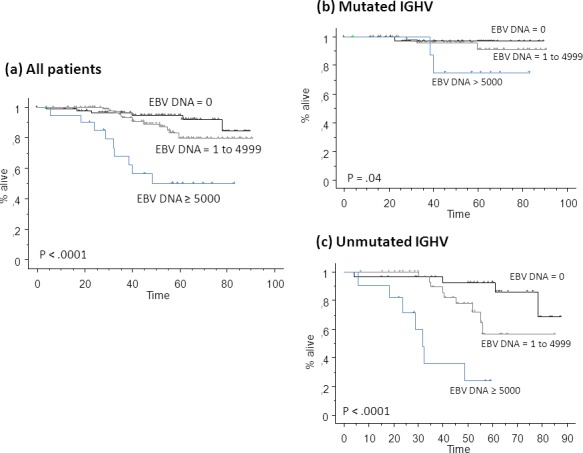
Kaplan Meier plot for overall survival (OS) of all patients from the learning set pooled on the basis of different EBV DNA loads **(a)** Patients with mutated immunoglobulin heavy chain variable region (*IGHV*) mutational status **(b)**, and patients with unmutated *IGHV* **(c)** are shown. Reported *P*-values refer to comparison between EBV DNA ≥ 5000 copies/µg DNA and EBV DNA = 0 copies/µg DNA. P-values between EBV DNA = 1 to 4999 and EBV DNA ≥ 5000 copies/µg DNA in patients with mutated and unmutated *IGHV* were .001 and .04, respectively. All other differences were not statistically significant.

As Cox model indicated that EBV DNA load was predictive as continuous variable, and given that EBV DNA load varied widely from patient to patient (range 0-36449), we analyzed the impact of increasing levels of EBV DNA load on OS. When patients were divided in three groups according to frequency distribution of EBV DNA load (10^th^ to 25^th^ *vs* 25^th^ to 90^th^ *vs* >90^th^ percentile), we observed a progressive impairment of OS curves (Figure 4). Of note, patients with EBV DNA load ≥5000 copies/µg DNA (21, 10%) had an extremely poor 5-years OS of 50% ± 12% *vs* 95% ± 3% for patients with EBV DNA load of 0 (P < .0001) *vs* 80% ± 5% for those with EBV DNA load of 1 to 4999. Again, as shown in Figure 4b-4c, results were independent of *IGHV* mutational status.

## DISCUSSION

In this study, we present for the first time an analysis on the clinical significance of EBV DNA load in patients with CLL at disease presentation. We have shown that EBV DNA load, tested in MNC, is detectable in more than half of patients, and that high levels (≥ 2000 copies/µg DNA) are found in one fifth of patients. We found that EBV DNA load had a strong relationship with OS. The predictive value of viral load was independent of commonly recognized prognostic factors, and increasing levels of EBV DNA load were directly associated to worse outcome. Patients with EBV DNA ≥ 2000 copies/µg DNA had a 5-years OS of 67% ± 8%, which resembled patients with del17p/*P53* gene mutation (5-years OS of 60% ± 16%) or patients with unmutated *IGHV* (5-years OS 69% ± 6%). An independent retrospective series of patients with longer follow-up corroborated our findings.

Our study has the merit of analysing a prospective series (learning set), avoiding bias related to patients selection. Despite the relatively short follow up for this indolent disease (54 months), the discriminating power of EBV DNA on OS appeared relevant. Since EBV replication is known to be related to severe immunosuppression, we hypothesized that the excess of deaths in patients with high EBV DNA load was due to infections or second tumors. However, deaths due to infections were 9% and 8% in patients with high or low EBV DNA load, respectively, while fatal second cancers were observed in 12% of patients with high EBV DNA and in 5% of patients with low EBV DNA load (P = .25). The poor prognosis conferred by high EBV DNA load was attributable, at least in part, to shorter TTFT (P = .0008), reflecting a more pronounced tumor aggressiveness, regardless of clinical stage at diagnosis (Binet A, B or C). Moreover, no significant relationship between EBV DNA load and age, or unmutated *IGHV* was observed. These findings suggest that EBV may not be a mere manifestation of the underlying immunosuppression associated with CLL, although further studies are needed to clarify this.

So far, molecular detection of EBV DNA in circulating MNC or plasma of patients with CLL had not been consistently reported in the literature. Hermouet et al. [20] reported that 11 of 21 patients (52%) with CLL had measurable EBV DNA load in peripheral blood lymphocytes by a real-time PCR assay. More recently, a study from Poland found detectable EBV-DNA load in mononuclear cells of 54% patients with CLL [21]. Similarly to our findings, an association between higher EBV load, CD38 expression and del11q, together with a shorter treatment-free survival was reported.

We acknowledge that our observations cannot define the biological role of EBV in CLL. Further studies are needed, as it has been done in EBV-driven lymphoproliferative disorders [3-5]. We observed that EBV DNA was detectable in sorted CLL B-lymphocytes in five patients at similar levels than in MNC, while it was consistently undetectable in the plasma of 20 patients with either high or low EBV DNA load. This indicates that EBV may latently infect CLL B-cells, since the absence of DNA in plasma samples argues against an active lytic infection. At our cut-off value (2000 copies/µg DNA), roughly one cell out of 100 is infected by the virus (1 µg of DNA corresponds to approximately 100.000 infected cells or 200.000 EBV DNA copies). However, since EBV does not behave as a normal gene, and each cell could contain more than one viral genome, 2000 copies/ug DNA may correspond to less than one infected cell every 100. Therefore, we can speculate that a relatively small number of CLL B-cells is infected by the virus at disease presentation, and that this EBV-related sub-clone would subsequently expand conditioning B-cells proliferation and the microenvironment. When latently infecting B lymphocytes EBV induces sustained telomerase activity [22], which is known to confer to B-CLL cases an aggressive clinical behaviour [23]. This link between EBV and telomerase may represent an explanation to the accelerated clinical course we have described in patients with high EBV DNA load. Longitudinal monitoring of EBV DNA load at different time points during CLL course would be of key importance to verify this theory. On the other hand, the presence of EBV DNA at higher levels in some patients may merely represent a consequence of the deeper immunosuppression, which is known to enhance tumor aggressiveness. For this reason, an assessment of T-lymphocyte and natural killer cell count, together with immunoglobulines level is warranted in these patients.

The literature does not provide a consistent threshold value corresponding to “high” EBV load, despite EBV is routinely investigated using sensitive and quantitative PCR methods, to relate virus loads to disease emergence and progression in several hematological malignancies including post-transplant lymphoproliferative disorders [24, 25]. We observed that 95% of our healthy subjects had EBV DNA load < 2000 copies/µg DNA, and we assumed this as a cut-off value to define high level. Similarly, Stevens et al. [19] found that MNC samples of healthy donors had EBV DNA load < 2000 copies/ml in almost all cases. This is consistent with previous studies that found EBV genome copy number in adults with latent EBV infection [24]. The variations in EBV DNA detection rates may be ascribed to the use of different assay systems, and to the lack of an agreed calibrator, specimen type, or unit adopted for reporting [25]. Obviating the need of a specific cut-off, EBV DNA load as a continuous variable was highly predictive in our series (Figure 4).

EBV has been well characterized in cases of Richter's transformation of CLL, particularly those displaying abundant Reed – Sternberg type cells [26]. In some, but not all cases, the malignant transformation occurs in cells originating from the CLL clone. In other cases of patients heavily pre-treated with T cell suppressants such as fludarabine, Richter's has been associated with the development of an unrelated EBV-associated B-cell clone showing that EBV or other viruses could drive Richter's transformation in some patients with CLL in the presence of impaired EBV-specific T-cell immunity [27, 14-16]. However, the precise role of EBV infection in Richter's transformation remains to be determined. In our series we described 12 patients developing Richter's transformation during follow-up (8 in the learning set and 4 in the validation set). Four of these had EBV DNA load ≥ 2000 copies/µg DNA (Table 1 and Table S1). Median EBV DNA load among patients with Richter's transformation was higher (1232 copies/µg DNA) than in patients without transformation (185 copies/µg DNA), but this difference was not statistically significant (P = .16). It is conceivable that sample collection in our patients undergoing later transformation was obtained too early, thus hampering a more precise evaluation of EBV role in driving CLL transformation.

In summary, our analysis on prospective patients with CLL indicates that EBV is present in peripheral MNC in more than half of patients with newly diagnosed CLL. High levels of EBV DNA are associated with an aggressive clinical behavior and short survival. The independence of EBV DNA load from most widely recognized biological and clinical prognostic factors indicates that a quantitative test for EBV DNA load could be integrated in the initial evaluation of patients to better define their outcome. Further studies are needed to clarify whether EBV has an active role in enhancing CLL progression or is merely a manifestation of the underlying immunosuppressed state associated with the disease.

## MATERIALS AND METHODS

### Patients

A total of 220 consecutive patients with newly diagnosed CLL referred to two major Hematology Divisions (San Bortolo Hospital, Vicenza, and University Hospital, Verona), were enrolled between June 2007 and December 2013, and followed prospectively. These patients constituted a learning set for prognostic considerations. A subsequent cohort was used for independent confirmation (validation set). In all patients biological material was collected at diagnosis, before receiving any cytotoxic treatment. All patients met the CLL diagnostic criteria of National Cancer Institute Working Group [18], and gave written informed consent to donate their blood and to have their clinical data collected into a dedicated database. Patients with monoclonal B-cell lymphocytosis (MBL) were excluded. The study was approved by the ethic review board of San Bortolo Hospital, as an ancillary study of the “CLL Veneto” project, which was started in 2007 as a prospective clinical and biological registry of incident patients diagnosed with CLL.

### Clinical characteristics and treatment

The median age of patients enrolled in the learning set was 65 years (range 30-86 years), with a male/female ratio of 1.97. Clinical and biological characteristics are listed in Table 1. At the time of this analysis, median follow-up from CLL diagnosis was 54 months (range 12-98). Overall, 81 patients (37%) received induction chemo-immunotherapy consisting of rituximab, fludarabine, cyclophosphamide in 36 (45%), rituximab and chlorambucil in 20 (25%), chlorambucil alone in 12 (14%), and other immunochemotherapies in 13 (16%), following the guidelines of the CLL Veneto protocol, which were based on widely accepted guidelines in terms of treatment and visits of follow-up [18].

### Blood processing, DNA and RNA extraction

At CLL diagnosis, we collected 20 mL of whole blood for each patient in EDTA-rinsed tubes. Plasma was obtained by centrifugation of samples. Peripheral blood MNC were isolated by Ficoll gradient, and were used for DNA and RNA extraction using QIAGEN spin-column kits and automatically extracted by QIAcube (Qiagen). Extracted DNA and RNA were then quantified by using the Nanodrop spectrophotometer (Thermo Scientific). Both plasma and nucleic acids were subsequently frozen at −80°C.

In five cases CLL B-cell subpopulations expressing CD19/CD5/CD23/CD20/CD43 were sorted by fluorescence-activated cell sorting (FACSAria II, Becton Dickinson, San Jose, CA) and DNA was extracted both from MNC and sorted B cells for comparison of EBV DNA load.

### Real time polymerase chain reaction (PCR) for measuring EBV DNA

EBV DNA load was assessed by quantitative PCR using DNA extracted from plasma or MNC, and was expressed as number of DNA copies per µg of total extracted DNA (copies/µg DNA). Analysis were made by setting up real time PCR performed on Rotor-Gene Q (Qiagen) using artus EBV QS-RGQ kit (Qiagen) following the instructions provided by the company. The EBV RGQ Master contains reagents and enzymes for the specific amplification of a 97 bp region of the EBNA-1 gene. An internal control and external positive controls were included to monitor the efficiency of sample preparation and downstream assay. Further details are available at http://www.qiagen.com/resources.

### Epstein-Barr virus serological pattern

The presence of serum immunoglobulin G (IgG) and IgM antibodies against the viral capsid antigen (VCA) was studied in 74 patients at CLL diagnosis using chemiluminescence immunoassay. Sixty-eight patients (92%) were IgG+/IgM, 4 (5%) were IgG+/IgM+, and 2 (3%) were IgG−/IgM−.

### Healthy subjects

Forty-one healthy subjects without signs or symptoms of recent or ongoing fever, no enlarged lymphnodes or infectious episodes were recruited among blood donors or volunteers, and their peripheral blood MNC were isolated tested for EBV DNA load. Sixteen were females and median age was 59 years old (range 32-74). We included 20 subjects older than 60 to minimize age-related bias in comparison to CLL patients.

### Validation set

For validation, we used an independent series of 112 patients with CLL, diagnosed at San Bortolo Hospital between January 2001 and May 2007. This set included retrospective cases with available stored biological material at CLL diagnosis and follow-up data. Median follow-up from CLL diagnosis was 103 months (range 14-168). Their clinical presentation and survival appeared representative to an unselected cohort of consecutive patients with CLL (Table S1) [18].

### Phenotypical, molecular, and genetic analysis

CD49d, ZAP-70 and CD38 expression were assessed on peripheral blood samples by flow cytometry, as previously described [28]. Extracted RNA was used to assess immunoglobulin heavy chain variable region (IGHV) mutational status, and stereotypes of the B-cell receptor were determined by analyzing the complete sequence of the heavy-chain complementary-determining region 3 in 210 patients (137 from the learning set and 73 from the validation set), as previously reported [29].

Cytogenetic abnormalities were evaluated by cytogenetic and fluorescence in situ hybridization (FISH) according to the hierarchical risk model of FISH anomalies [30]. The mutation hot spots of the TP53 (exons 4-9, including splicing sites; RefSeq NM_000546.5), NOTCH1 (exon 34; including splicing sites; RefSeq NM_017617.2), SF3B1 (exons 14, 15, 16, 18, including splice sites; RefSeq NM_012433.2), MYD88 (exons 3, 5, including splicing sites; RefSeq NM_002468.4), and BIRC3 (exons 6-9, including splicing sites; RefSeq NM_001165.4) genes were analyzed on extracted DNA by PCR amplification and DNA direct sequencing of high-molecular-weight genomic DNA, as previously described [31].

### Statistical analysis

EBV DNA load ≥ 2000 copies/µg DNA, corresponding to the 95^th^ percentile of healthy subjects, was defined as high value, in keeping with data of the literature [19].

Overall survival (OS), defined as the time interval between CLL diagnosis and last follow-up or death for any cause, was considered the primary outcome of the study. Time-to-first-treatment (TTFT) was defined as the time interval between the date of presentation and date of first CLL treatment. Patients who did not receive any treatment during follow-up were censored at their last follow-up date. Actuarial OS and TTFT were estimated by the Kaplan-Meier method [32] and differences were analyzed by the log rank test. Proportional Cox Hazard Model was used for multivariate analysis [33].

The comparison of clinical and biological features between patients with high *vs* low load was carried out with the χ2 test or with the nonparametric Mann-Whitney test, as appropriate. The Kolmogorov–Smirnov test (K–S test) was used to define the distribution of the EBV DNA load as continuous variable. All variables found to have a P value less than or equal to .05 were considered to be statistically significant. All statistical calculations were performed using StatView (Abacus Concepts, Berkeley, CA).

## SUPPLEMENTARY FIGURE AND TABLE


