# Supplementary material for: Epstein-Barr virus DNA load in chronic lymphocytic leukemia is an independent predictor of clinical course and survival
Source: Oncotarget. 2015 Jun 10;6(21):18653–63. doi: 10.18632/oncotarget.4418 (PMC4621917; doi:10.18632/oncotarget.4418)
Supplement: Supplementary file 1 [file oncotarget-06-18653-s001.pdf]

Epstein-Barr virus DNA load in chronic lymphocytic leukemia is an independent predictor of clinical course and survival

Supplementary Material

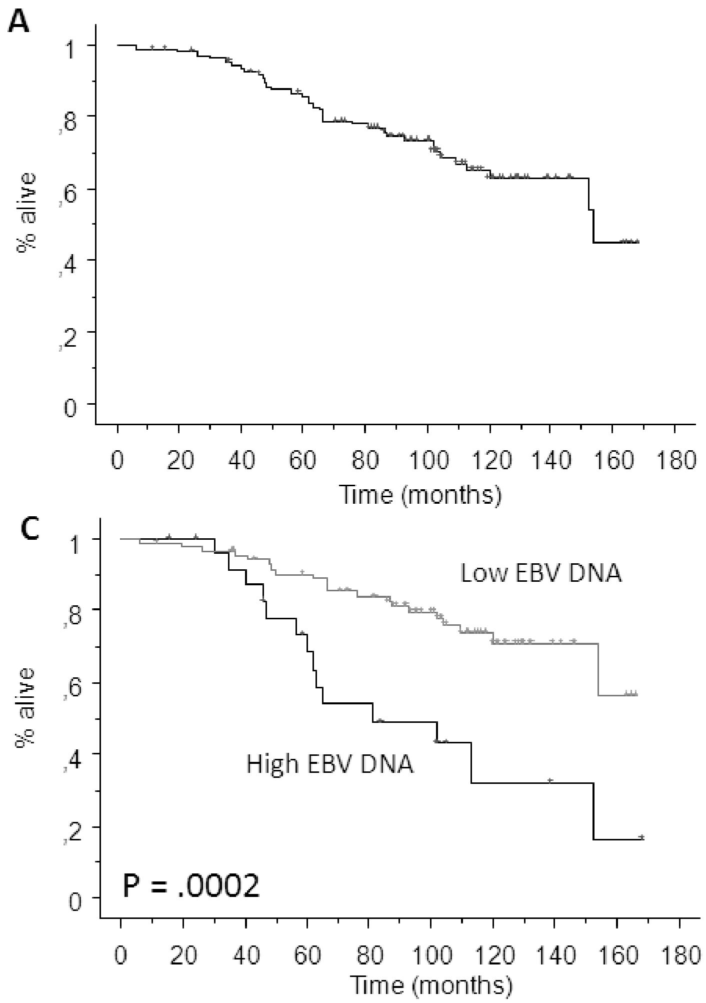

Supplementary Figure 1. Kaplan Meier plot for OS (A) and TTFT (B) of patients from the validation set, dichotomized according to high or low EBV DNA load (≥ vs < 2000 copies/µg DNA, C and D). Hazard ratio and 95% confidence interval were 3.363 (1.698-6.761) and 1.972 (1.127-3.505) for OS and TTFT, respectively.

Supplementary Table 1. Clinical and biological characteristics of 112 patients of the validation cohort with chronic lymphocytic leukemia at disease presentation, then divided according to EBV DNA viral load (copies/µg of DNA).

	**All pts** *N* = 112	**EBV DNA ≥ 2000** *N* = 25	**EBV DNA < 2000** *N* =87	***P*-value**
**Median Age**, years (range)	63 (34-91)	66 (42-84)	61 (34-91)	0.10*
**Female gender**	45 (40%)	10 (40%)	35 (40%)	0.98
**Median lymphocyte count**, x10^3/mmc (range)	9.6 (2.6-440)	8.9 (3.1-90)	9.8 (2.6-440)	0.38*
**Richter's transformation**	4 (4%)	1 (4%)	3 (3%)	0.90
**Second cancer**	5 (5%)	1 (4%)	4 (5%)	0.90
**BINET stage**				
A	92 (82%)	20 (80%)	72 (83%)	0.75
B	16 (14%)	4 (16%)	12 (14%)	0.78
C	4 (4%)	1 (4%)	3 (3%)	0.89
**FISH°**				
Normal	16 (37%)	3 (27%)	13 (41%)	0.42
del13q	12 (28%)	2 (18%)	10 (31%)	0.40
12+	5 (12%)	2 (18%)	3 (9%)	0.43
del11q	5 (12%)	2 (18%)	3 (9%)	0.43
del17p	3 (7%)	1 (9%)	2 (6%)	0.74
**IGHV mutational status°**				
Unmutated IGHV	41 (38%)	16 (64%)	25 (30%)	**0.002**
**Immunophenotype**				
CD38+	27 (27%)	10 (50%)	17 (21%)	**0.009**
ZAP70+°	33 (37%)	9 (53%)	24 (33%)	0.13

Abbreviations: FISH: fluorescence in situ hybridization according to the hierarchical risk model; del13q: deletion in chromosome 13q14; del11q: deletion in chromosome 11q23; del17p: deletion in chromosome 17p12; +12: trisomy 12; IGHV: immunoglobulin heavy chain variable region genes. *Calculated with the Mann-Whitney test; °FISH available in 43 patients, IGHV mutational status in 105, immunophenotype for CD38 100, ZAP70 in 89.
